# The crystal structure of the putative peptide-binding fragment from the human Hsp40 protein Hdj1

**DOI:** 10.1186/1472-6807-8-3

**Published:** 2008-01-22

**Authors:** Junbin Hu, Yunkun Wu, Jingzhi Li, Xinguo Qian, Zhengqing Fu, Bingdong Sha

**Affiliations:** 1 Department of Cell Biology, University of Alabama at Birmingham, Birmingham, AL 35294, USA; 2 SER-CAT, APS, Argonne National Laboratory, 9700 S. Cass Ave., Argonne, IL 60439, USA

## Abstract

**Background:**

The mechanism by which Hsp40 and other molecular chaperones recognize and interact with non-native polypeptides is a fundamental question. How Hsp40 co-operates with Hsp70 to facilitate protein folding is not well understood. To investigate the mechanisms, we determined the crystal structure of the putative peptide-binding fragment of Hdj1, a human member of the type II Hsp40 family.

**Results:**

The 2.7Å structure reveals that Hdj1 forms a homodimer in the crystal by a crystallographic two-fold axis. The Hdj1 dimer has a U-shaped architecture and a large cleft is formed between the two elongated monomers. When compared with another Hsp40 Sis1 structure, the domain I of Hdj1 is rotated by 7.1 degree from the main body of the molecule, which makes the cleft between the two Hdj1 monomers smaller that that of Sis1.

**Conclusion:**

This structural observation indicates that the domain I of Hsp40 may possess significant flexibility. This flexibility may be important for Hsp40 to regulate the size of the cleft. We propose an "anchoring and docking" model for Hsp40 to utilize the flexibility of domain I to interact with non-native polypeptides and transfer them to Hsp70.

## Background

Molecular chaperones are a large group of proteins that can bind and stabilize non-native polypeptides and facilitate protein folding into their native conformations [1-3]. Heat shock protein 70s (Hsp70s) play essential roles in cell physiology and have been well studied [1-3]. Members of the Hsp70 and Hsp40 (DnaJ-like) families function in specific pairs that form transient complexes with non-native regions of polypeptides to promote the folding, assembly and transport of proteins within the cell [1-7].

All types of Hsp40 proteins contain a J-domain that can stimulate the ATPase activities of Hsp70. Both type I and type II Hsp40s have a peptide-binding fragment located at the C-terminus of the proteins. The N-terminal J-domains are connected to the peptide-binding fragments via a G/F rich linker in both type I and type II Hsp40s. Type I Hsp40 such as *E. coli *DnaJ, yeast Ydj1 and human Hdj2 contain two Zinc-finger motifs between the J-domain and the C-terminal peptide-binding fragment while type II Hsp40 proteins such as human Hdj1 and yeast Sis1 do not [8-10]. It was proposed that Hsp40 binds non-native polypeptide first and then delivers the non-native polypeptide to Hsp70 for folding [1,11,12]. The ability to bind non-native polypeptides for the cytosolic Hsp40 is an essential function *in vivo *[13].

The crystal structures of the peptide-binding fragments of Ydj1 and Sis1, the type I and II Hsp40 proteins from *S. cerevisiae*, was determined to 2.7 Å resolution, respectively [14-17]. The crystal structures revealed that both Ydj1 and Sis1 peptide-binding fragments functioned as a homo-dimer with a large cleft between the two elongated monomers. The Sis1 monomer contains domain I, II and a short C-terminal dimerization motif. A hydrophobic depression was revealed as the putative peptide-binding site on the molecular surface of the domain I of Sis1 monomer [14,17]. In the crystal structure of Ydj1 complexed with the peptide substrate, the domain I and domain III of Ydj1 resemble the structure of domain I and II of Sis1 while two Zinc finger motifs form the domain II of Ydj1 [15,16]. The peptide substrate binds to the domain I of Ydj1 and form a β-strand with the Ydj1 molecule. The side chains of the peptide substrate make extensive hydrophobic interactions with a hydrophobic pocket located on domain I of Ydj1.

It has been showed that the extreme C-terminal four amino acid residues EEVD within human Hsp70 play regulatory roles in Hsp40/Hsp70 functions [4]. Deletion of these four residues compromised the protein refolding capability of human Hsp70 facilitated by the human Hsp40 Hdj1 [4,6]. The crystal structure of Sis1 peptide-binding fragment complexed with the Hsp70 Ssa1 C-terminus has been determined [7]. The Ssa1 extreme C-terminal eight amino acid residues GPTVEEVD form a β-strand with the domain I of Sis1 peptide-binding fragment. The Ssa1 C-terminus binds Sis1 at the site where Sis1 interacts with the non-native polypeptides.

The human type II Hsp40 Hdj1 has been shown to function as a molecular chaperone to promote efficient protein folding. Hdj1 can bind to the non-native polypeptide and suppress the protein aggregation. Hdj1 can also pair with Hsp70 to refold the non-native polypeptides [4,18]. Both of human Hdj1 and yeast Sis1 belong to type II Hsp40 family and they might have similar *in vivo *functions [19]. The crystal structure of Hdj1 presented in this paper may reveal the mechanism by which Hdj1 functions as a molecular chaperone to promote protein folding.

## Results

### Structure determination of the human Hsp40 Hdj1 putative peptide-binding fragment

We have expressed and purified the human type II Hsp40 Hdj1 putative peptide-binding fragment (residues 158–340). The protein was then crystallized by use of hanging drop vapor diffusion method. The crystal structure of the Hdj1 putative peptide-binding fragment was determined to 2.7Å resolution by the molecular replacement method using the Sis1 structure as the search model (Table I). The resultant electron density map from the molecular replacement method allowed us to trace the residues 162 to 335 of Hdj1 except for a short loop region (residues 228 to 230).

**Table 1:** Statistics for Hdj1 data collection and structure determination (Numbers in parenthesis are for the outer resolution shell.)

	**Hdj1 fragment**
**Data collection**	
Resolution (Å)	2.7 (2.81-2.70)
*R*_sym_	0.065(0.382)
*I*/σ*I*	18.1(2.1)
Completeness (%)	89.8%(65.9%)
Redundancy	5.7
	
**Refinement**	
Resolution (Å)	30.0-2.7
No. reflections	9943 (681 used for R_free _calculation)
*R*_work_	28.3 (38.8)
*R*_free_	33.6 (42.3)
R.m.s deviations	
Bond lengths (Å)	0.01
Bond angles (°)	1.50
Impropers (°)	1.09
Dihedrals (°)	25.8

**Figure 1:**
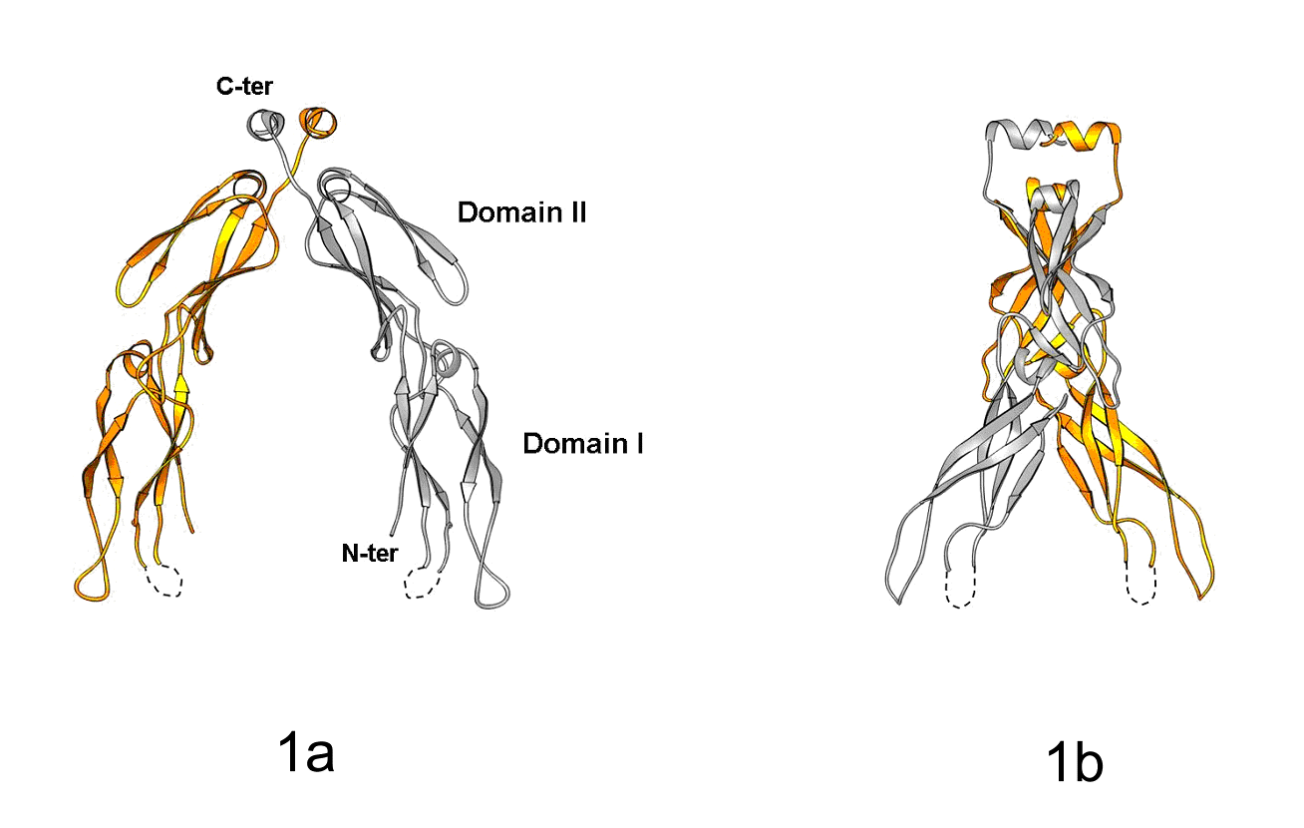
The crystal structure of the Hdj1 putative peptide-binding fragment. ***a) ***Ribbon drawing of the Hdj1 putative peptide-binding fragment homo-dimer [27]. One monomer of the Hdj1 is shown in silver and the other monomer is shown in gold. The two monomers are related by a vertical two-fold axis. The missing short loop region (residues 228 to 230) is indicated by a dotted line. Domain I and II of Hdj1 are labeled. The N-terminus and C-terminus of Hdj1 are also labeled. ***b) ***A view of Hdj1 dimer after it is rotated 90° along the vertical axis from the orientation shown in Fig. 1*a*.

Hdj1 putative peptide-binding fragment forms a dimer in the crystal structure. The two monomers are related by a crystallographic two-fold axis. The structure of the Hdj1 (156–340) monomer consists of eleven β-strands (B1-B11) and three short α-helixes (A1-A3; Fig. 1). The Hdj1 monomer structure contains domain I, II and the C-terminal dimerization motif. Both domain I and II have a core formed by a major β-sheet and a minor β-sheet that are connected by a short helix. The two Hdj1 monomers are associated into a homo-dimer through the C-terminal dimerization motif (Fig. 1). The Hdj1 homo-dimer forms a U-shaped architecture and a large cleft is formed between the two elongated monomers.

### The comparison between Human Hsp40 Hdj1 and yeast Hsp40 Sis1 structures

**Figure 2:**
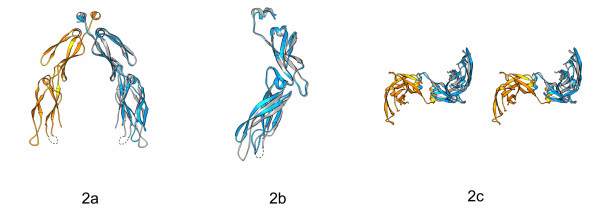
The structural comparison between Hdj1 and Sis1 Structures in Ribbon drawings. ***a) ***The Hdj1 structure is superimposed with the Sis1 structure after the domain II of Hdj1 is aligned with that in the Sis1. The color definition of Hdj1 homo-dimer is the same as that in Fig. 1. Sis1 structure is in blue. The Hdj1 dimer is shown while only the Sis1 monomer is drawn in this figure. ***b) ***A view of Hdj1 and Sis1 structures after they are rotated 90° along the vertical axis from the orientation shown in Fig. 2*a*. Only Hdj1 and Sis1 monomer structures are shown in this figure for clarity. ***c) ***A stereo view of Hdj1 and Sis1 structures after they are rotated 90° along the horizontal axis from the orientation shown in Fig. 2a. The cleft faces readers. The Hdj1 dimer is shown while only the Sis1 monomer is drawn in this figure.

We have previously determined the crystal structure of yeast type II Hsp40 Sis1 peptide-binding fragment at 2.7 Å resolution [14]. Therefore, it is of interest to compare these two Hsp40 protein structures from different species (Fig. 2). Both Hdj1 and Sis1 monomer structures contain domain I, domain II and the C-terminal dimerization motif. The individual domains from Hdj1 and Sis1 structure share similar folds. The Hdj1 dimer and Sis1 dimer have a similar U-shaped molecular shape with a large cleft between the monomers (Fig. 2).

However, significant differences exist between Hdj1 and Sis1 structures. When the domain II of Hdj11 is superimposed with that of the Sis1 structure, we found that the domain I of Hdj1 in the complex structure was kinked about 7.1° away from that in Sis1 structure (Fig. 2). The domain re-arrangements within Hdj1 were achieved by the rotation of the linker region between domain I and domain II (residues 242 to 249).

The domain arrangements between domain I and II of Hdj1 cause the two domain Is within the Hdj1 dimer closer to each other when compared with Sis1 structure (Fig. 2). This makes the cleft between two monomers within Hdj1 smaller than that of Sis1. The relative positions of the domain IIs and the C-terminal dimerization motifs within the Hdj1 and Sis1 homo-dimers, however, are kept almost the same when superimposed (Fig. 2). The structural differences between Hdj1 and Sis1 structures indicate that the domain I of Hsp40 may possess significant flexibility. This flexibility may be important for Hsp40 to interact with non-native polypeptides and transfer them to Hsp70.

**Figure 3:**
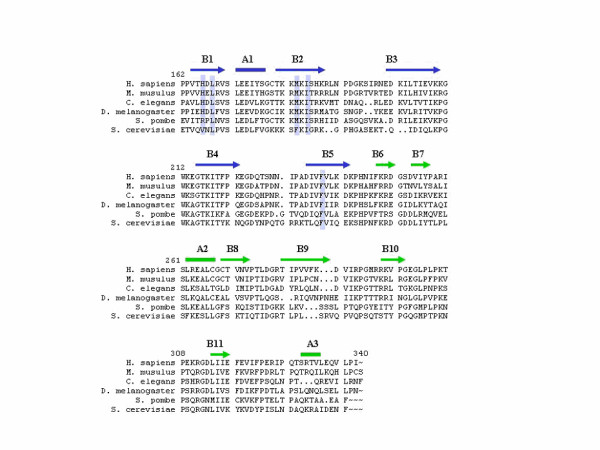
Sequence alignment of the C-terminal regions from eukaryotic Type II Hsp40 family members. Program Pileup from GCG package was utilized to align residues 162–340 of Hdj1 from *H. sapiens *with similar regions of Hsp40 proteins from *M. musculus *(Hsp40-3), *C. elegans *(Z66513.1), *D. Melanogaster *(Droj-1), *S. pombe *(Psi protein) and *S. cerevisiae *(Sis1). The amino acid residue numbers of Hdj1 are numbered above the alignment. The residues involved in forming the peptide-binding site of Hdj1 are marked by blue bars. The secondary structures of Hdj1 are shown on top of the alignment. The structural components in domain I are denoted by blue and those in domain II are denoted by green. The α-helices are represented by boxes and β-strands are represented by arrows.

Another major difference between Hdj1 and Sis1 structure is at the tip region of the domain I. The sequence alignment indicates that human Hsp40 Hdj1 has five more amino acid residues between β-strand B2 and B3 than Sis1 from yeast, while Hdj1 has one fewer residue between β-strand B4 and B5 than Sis1 (Fig. 3). When the domain Is from Hdj1 and Sis1 structures are superimposed, we found that the loop between B2 and B3 in Hdj1 structure protrudes significantly further away than that in Sis1 structure. The longer loop region at the tip of domain I in the Hdj1 structure helps to constitute a larger domain I than that in Sis1 structure. Sequence alignment shows that the loop region between B2 and B3 is longest for human and mouse type II Hsp40 among all species. It is two amino acid residues shorter in *C. elegans *and *Drosophila *and five amino acid residues shorter in yeast *S. cerevisiae *(Fig. 3).

On the other hand, the loop region between B4 and B5 in Hdj1 is one residue shorter than that in Sis1 structure (Fig. 3). Because part of the electron density is missing for this loop region in Hdj1 structure, it is difficult for us to compare this region between Hdj1 and Sis1 structure. The sequence alignment indicates that only Sis1 from *S. cerevisiae *has the longer loop between B4 and B5. All the other type II Hsp40s, however, contain the shorter version of the loop between B4 and B5.

### The peptide-binding site of human Hsp40 Hdj1

**Figure 4:**
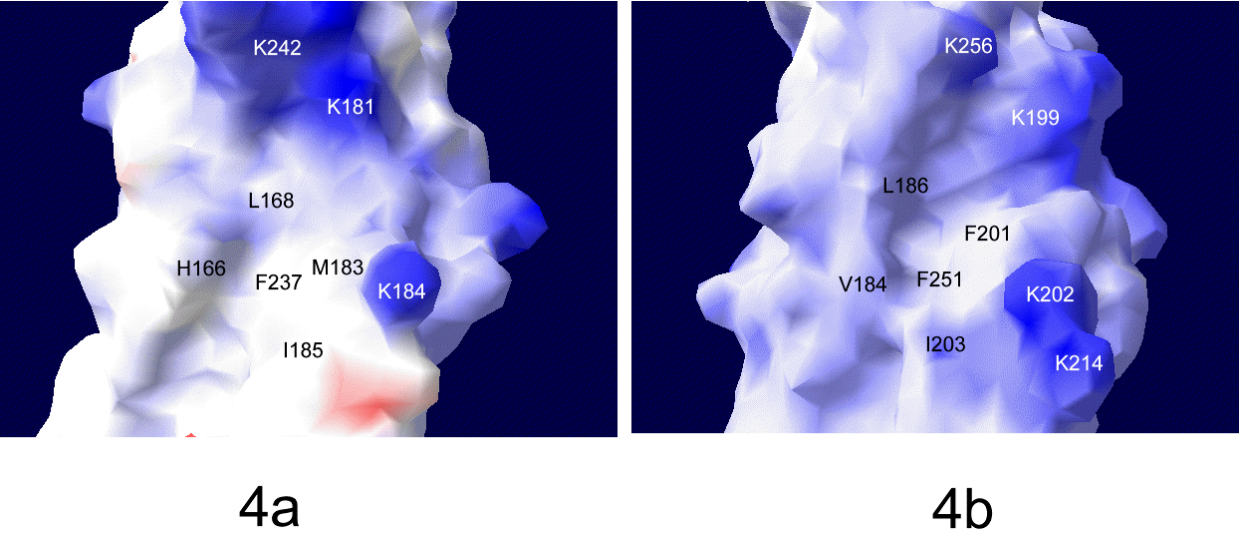
The surface potential drawings around the Hdj1 and Sis1 peptide-binding sites determined by Swiss-PDBviewer. Blue and red denote positively and negatively charged regions, respectively. ***a) ***Surface potential drawing presentation of the Hdj1 peptide-binding site. The residues of Hdj1 involved in forming the peptide-binding site are labeled in black. The Lysine residues involved in binding the Hsp70 C-terminal EEVD motifs are labeled in white. ***b) ***Surface potential drawing presentation of the Sis1 peptide-binding site.

Molecular chaperone Hsp40 can interact and stabilize the non-native polypeptides and prevent them from forming aggregations. The peptide-binding sites of both type I and type II Hsp40s for the non-native polypeptides have been located on the molecular surface of domain I. The Hsp40s may bind the non-native polypeptides through hydrophobic interactions [14-16]. When we examined the peptide-binding site on the domain I of Hdj1 structure, several hydrophobic residues were identified to participate in forming the peptide-binding site. These residues include L168, M183, I185 and F237 (Fig. 4). The hydrophobicity of these residues is well conserved among all species. However, a polar residue, H166, was also identified to be involved in constituting the peptide-binding pocket (Fig. 4). This Histidine residue is conserved for type II Hsp40s among all species except for yeast as shown in sequence alignment (Fig. 3). We reason that H166 may play an important role in positioning the side chains from Pro, Phe or Tyr residues through van der waals interactions.

Hsp40 and Hsp70 form transient complex to facilitate the efficient protein folding process [4,6]. It has been reported that Hsp40 may interact with the Hsp70 C-terminal EEVD motif by use of the peptide-binding site located on Hsp40 domain I [7]. The crystal structure of yeast Hsp40 Sis1 and Hsp70 Ssa1 C-terminus complex indicated that several Lysine residues (K199, K202, K214 and K256) in Sis1 are involved in binding the Hsp70 C-terminal EEVD motif by charge-charge interactions. The sequence alignment shows that the corresponding residues in Hdj1 are K181, K184, R198 and K242. In the Hdj1 structure, K181, K184 and K242 are well-positioned to interact with the EEVD motif from Hsp70 (Fig. 4). K181 and K242 are stabilized by forming salt bridges with a conserved E174. R198, however, points to the opposite direction from the peptide-binding site of Hdj1 and it is not likely to be involved in binding Hsp70 EEVD. K181, K184 and K242 are all conserved for type II Hsp40 among all species. Therefore these positively charged residues may be the common residues for type II Hsp40s to interact with the Hsp70 C-terminal EEVD motifs.

## Discussion

We report the crystal structure of human type II Hsp40 Hdj1 putative peptide-binding fragment to 2.7Å resolution. The structure reveals that Hdj1 forms a homo-dimer in the crystal by a crystallographic two-fold axis. The Hdj1 dimer has a U-shaped architecture and a large cleft is formed between the two elongated monomers. The human Hdj1 structure is similar to the *S. cerevisiae *Hsp40 Sis1, which may account for the fact that Hdj1 and Sis1 share similar *in vivo *functions [19]. However, the two structures have significant differences. When compared with yeast type II Hsp40 Sis1 structure, the domain I of Hdj1 shows a 7.1° rotation from the main body of the molecule, which makes the cleft between two Hdj1 monomers smaller. In the crystal structure of Hdj1, a polar residue H166 is involved in forming the peptide-binding site, which is different from the hydrophobic peptide-binding depression revealed in Sis1 structure. In addition, at the tip of domain I in Hdj1 structure, Hdj1 structure has a longer loop region than Sis1, which accounts for a larger domain I in Hdj1 structure.

The domain re-arrangement between domain I and domain II within type II Hsp40 has been observed when yeast Hsp40 Sis1 binds the Hsp70 Ssa1 C-terminus. Little conformational changes occur within the individual domain I and domain II of Sis1 after binding to Ssa1. However, we found that the domain I of Sis1 in the complex structure was kinked about 8.5° away from that in Sis1 structure. These conformational changes widen up the cleft between the two Sis1 monomers within the homo-dimer, where might be the docking site for Ssa1 peptide-binding domain [7]. In contrast, when we compare the crystal structures of Hdj1 and Sis1 peptide-binding fragment, we found that the domain Is within Hdj1 were swung towards each other by 7.1° from that in Sis1 structure, which makes the cleft between the two monomers smaller. These domain re-arrangements were achieved by the rotation of the linker region between domain I and domain II within both Sis1 and Hdj1 structures. The relative positions of the domain IIs within the Sis1 and Hdj1 dimer, however, are kept almost the same.

These structural observations indicate that domain I of Hsp40 may possess significant flexibility to take different conformations for its molecular chaperone activity. The flexibility in Sis1 could be important for the Hsp40/Hsp70 system to function. Previous studies have shown that the mutations within the interface of domain I and domain II in Sis1 can abolish the binding capability of Sis1 to Ssa1 [20]. It is possible that the conformational changes of Sis1 can not be achieved within these mutants to locate the domain I of Sis1 into the Ssa1 binding position.

In the Sis1 structure, the peptide-binding site is occupied by a Pro residue from a neighbor molecule. It is possible that the Sis1 structure may represent the conformation for Sis1 to bind the non-native polypeptide. The Sis1-Ssa1 complex structure may represent the conformation for Sis1 to interact with Hsp70 Ssa1. The peptide-binding site in the Hdj1 structure is not occupied by any amino acid residue, so the Hdj1 structure may represent the free Hsp40 state. Thus, Hsp40 may take different conformations in these three states.

**Figure 5:**
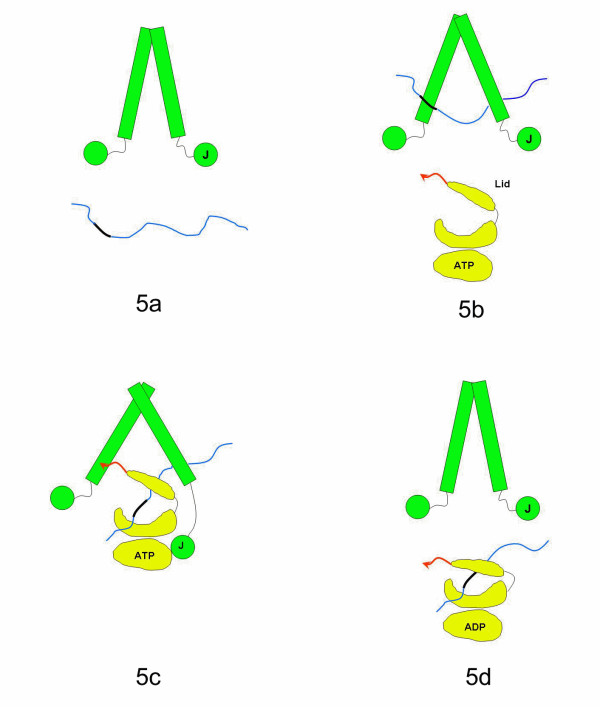
Schematic representation of the "anchoring and docking" mechanism by which Hsp40 (green) delivers a non-native peptide (blue) to Hsp70 (yellow). The cartoon drawing depicts an Hsp70 molecule that is divided into its ATPase domain, peptide-binding groove and the lid domain. The J-domain and peptide-binding fragment of Hsp40 are shown schematically. The C-terminus "anchor" region of Hsp70 is shown in a red arrow. The blue line denotes the extended non-native polypeptide. The thick black region in the non-native polypeptide indicates the hydrophobic region that can be recognized by Hsp40 peptide-binding fragment and Hsp70 peptide-binding domain. ***a) ***When Hsp40 is free of the non-native polypeptide, the cleft between the two monomers is narrow. ***b) ***The cleft is widened up when Hsp40 binds the non-native polypeptide. This may help to stretch the polypeptides into the extended conformations. ***c) ***The cleft within Hsp40 is further opened up when the Hsp70 C-terminus interacts with the Hsp40. ***d) ***When the Hsp70-non-native polypeptide complex is released from Hsp40, the cleft within Hsp40 returns to the narrow state.

An "anchoring and docking" model for Hsp70 to interact with Hsp40 Sis1 has been proposed, in which the Hsp70 C-terminus may function as an anchor to bind to Hsp40 [6,7]. The studies in this paper may add significant more details into the model (Fig. 5). When the Hsp40 is free of non-native polypeptides, it may keep a relatively small cleft within the dimer structure. The cleft may be widened up when Hsp40 binds the non-native polypeptide. The Hsp70 C-terminal anchor region may bind the Sis1 peptide-binding pocket and replace hydrophobic side chains from the non-native polypeptide. This may further widen up the cleft within Hsp40 homo-dimer. The freed non-native polypeptide may be bound by Hsp70 for subsequent protein folding (Fig. 5).

The low resolution quaternary structures of full-length type I and type II Hsp40s have been determined by small angle X-ray scattering [21]. The small angel X-ray scattering studies indicated that the two J-domains within the type II Hsp40 dimer are positioned at two opposite ends. The relative positioning of Hsp70 N-terminal domain and C-terminal domain has also been revealed by X-ray crystallography [22]. Our working model is consistent with the results from previous structural studies.

It is not very clear what roles that the Hsp40 conformational changes may play when binding the non-native polypeptides in Hsp40/Hsp70 molecular chaperone functions. Several possibilities may exist. 1. Widening up the cleft while Hsp40 interacts with non-native polypeptides may help to stretch the polypeptides into extended conformations. Hsp70 prefers to bind the polypeptides in extended conformations. 2. The enlarged cleft within the Hsp40 homo-dimer may provide more space for Hsp70 to dock and to interact with the non-native polypeptides.

## Conclusion

We have determined the crystal structure of the putative peptide-binding fragment of Hdj1, a human member of the type II Hsp40 family. The 2.7Å structure reveals that Hdj1 forms a homodimer in the crystal by a crystallographic two-fold axis. The Hdj1 dimer has a U-shaped architecture and a large cleft is formed between the two elongated monomers.

This structural observation indicates that the domain I of Hsp40 may possess significant flexibility. This flexibility may be important for Hsp40 to regulate the size of the cleft. We propose an "anchoring and docking" model for Hsp40 to utilize the flexibility of domain I to interact with non-native polypeptides and transfer them to Hsp70.

## Methods

### Crystallization of the Hdj1 putative peptide-binding fragment

We have cloned the human type II Hsp40 Hdj1 putative peptide-binding fragment (residues 158–340). The recombinant protein was then expressed in *E. coli *and purified by use of a Nickel-chelating column followed by a gel filtration column Superdex-75 (GE Healthcare). The protein was concentrated to 25 mg/ml in 10 mM Tris buffer (pH 8.0), NaCl 50 mM. The Hdj1 putative peptide-binding fragment was crystallized by use of hanging drop vapor diffusion method with the mother liquid of 100 mM Citric acid buffer (pH 5.5), 25% PEG400 at room temperature.

### Structure determination and refinement

The diffraction data for the Hdj1 crystals were collect at APS beamline SERCAT. The crystals were flash frozen at 100 K in a nitrogen gas stream in the cryoprotectant consisting of 100 mM citric acid buffer (pH 5.5), 35% PEG400. The crystals diffracted to 2.7Å using the X-ray at 1.0Å wavelength. The data were processed by HKL2000 package. The crystals belong to the space group of C222_1 _with the cell parameters of a = 97.01Å, b = 191.13 and c = 40.96Å.

Program EPMR through the package SGXPRO was utilized to carry out the molecular replacement method [23]. The Sis1 peptide-binding fragment monomer structure was used as the searching model. The sequence identity between Sis1 and Hdj1 peptide-binding fragment is 33%. One clear solution was revealed through the search. The resultant electron density map allowed us to carry out the molecular tracing by use of the program O [24]. The residues 162 to 335 of Hdj1 except for a short loop region (residues 228 to 230) were modeled into the electron density map.

The model was then refined by program CNS against the 2.7Å data collected at APS [25]. One cycle of temperature annealing at 2000 K and six cycles of positional refinement were then carried out. Restrained individual B-factor refinement was not performed until the last cycle. After each cycle of refinement, the model was manually rebuilt according to the resultant 2Fo-Fc and Fo-Fc maps. The refinement gave reasonable R.M.S. derivation from the ideal geometry at this resolution (Table 1). The possible bias for Hdj1 structure determination generated by using Sis1 as a model is not very likely because we see clear side chains for Hdj1 molecule in the resultant electron density map. The R_free _is also within the reasonable range, which indicates the Hdj1 structure is correct.

A Ramachandran plot of the final model by use of program Probity revealed that 87.7% of the residues in the structure were in the favored regions and 97.2% of the residues were in allowed regions [26].

#### Coordinates

The coordinates and structure factors of Hdj1 putative peptide-binding fragment crystals have been deposited to Protein Data Bank with an accession number of 2QLD.

## Authors' contributions

JH carried out the protein expression, purification and crystallization. JH also contributed to data collection and structure determination. YW contributed to data collection and structure determination. JL contributed to protein expression. ZF contributed to structure determination. BDS participated in structure determination and manuscript preparation. All authors read and approved the final manuscript.
